# Outcomes After Liver Transplantation With Incidental Cholangiocarcinoma

**DOI:** 10.3389/ti.2022.10802

**Published:** 2022-11-02

**Authors:** Nawaz Z. Safdar, Abdul R. Hakeem, Rosemary Faulkes, Fiona James, Lisa Mason, Steven Masson, James Powell, Ian Rowe, Shishir Shetty, Rebecca Jones, Harry V. M. Spiers, Neil Halliday, Jack Baker, Douglas Thorburn, Raj Prasad, Richard Parker

**Affiliations:** ^1^ School of Medicine, University of Leeds, Leeds, United Kingdom; ^2^ Leeds Liver Unit, St James’ University Hospital, Leeds, United Kingdom; ^3^ University Hospitals Birmingham, Birmingham, United Kingdom; ^4^ Royal Infirmary of Edinburgh, Edinburgh, United Kingdom; ^5^ Newcastle Upon Tyne Hospitals, Newcastle, United Kingdom; ^6^ Roy Calne Transplant Unit, Addenbrooke’s Hospital, Cambridge, United Kingdom; ^7^ The Royal Free, London, United Kingdom

**Keywords:** survival, tumor, intrahepatic, hilar, indication, transplant, distal, hepatocellular

## Abstract

Cholangiocarcinoma (CCA) is currently a contraindication to liver transplantation (LT) in the United Kingdom (UK). Incidental CCA occurs rarely in some patients undergoing LT. We report on retrospective outcomes of patients with incidental CCA from six UK LT centres. Cases were identified from pathology records. Data regarding tumour characteristics and post-transplant survival were collected. CCA was classified by TNM staging and anatomical location. 95 patients who underwent LT between 1988–2020 were identified. Median follow-up after LT was 2.1 years (14 days-18.6 years). Most patients were male (68.4%), median age at LT was 53 (IQR 46-62), and the majority had underlying PSC (61%). Overall median survival after LT was 4.4 years. Survival differed by tumour site: 1-, 3-, and 5-year estimated survival was 82.1%, 68.7%, and 57.1%, respectively, in intrahepatic CCA (*n* = 40) and 58.5%, 42.6%, and 30.2% in perihilar CCA (*n* = 42; *p* = 0.06). 1-, 3-, and 5-year estimated survival was 95.8%, 86.5%, and 80.6%, respectively, in pT1 tumours (28.2% of cohort), and 65.8%, 44.7%, and 31.1%, respectively, in pT2-4 (*p* = 0.018). Survival after LT for recipients with incidental CCA is inferior compared to usual outcomes for LT in the United Kingdom. LT for earlier stage CCA has similar survival to LT for hepatocellular cancer, and intrahepatic CCAs have better survival compared to perihilar CCAs. These observations may support LT for CCA in selected cases.

## Graphical Abstract

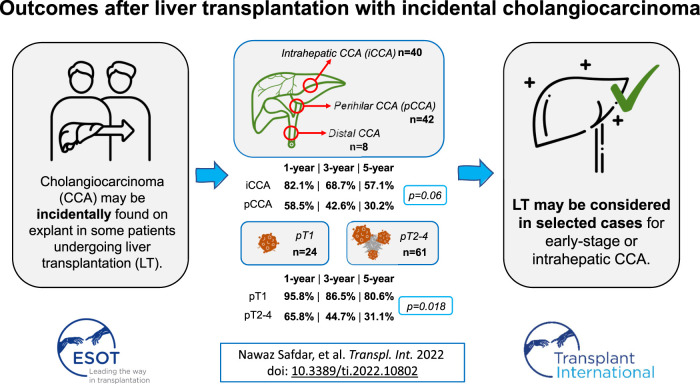

## Introduction

Cholangiocarcinoma (CCA) is the second most common primary hepatic malignancy, with increasing age-standardised incidence rate in England from 2.7 in 2001 to 4.3 per 100,000 in 2017 (1). Despite improvement in diagnostic tools, chemotherapeutic agents, and surgical techniques, the age-standardised mortality rate has increased from 2.6 in 2001 to 4.7 per 100,000 in 2017 (2, 3). Primary sclerosing cholangitis (PSC) is a known risk factor for the development of CCA (4): individuals diagnosed with PSC have a 15%–20% lifetime risk of developing CCA (5). A meta-analysis of 11 studies found that cirrhosis, hepatitis B, hepatitis C, alcohol, diabetes and obesity are also risk factors for development of CCA (6).

Diagnosis of CCA can be extremely challenging, as a significant proportion of patients do not present with definite mass lesions. Although intrahepatic CCA (iCCA) can be visualised as mass-forming lesions (7), perihilar CCA (pCCA) and distal CCA (dCCA) can be tricky to detect due to their infiltrative nature (8). Difficulties with diagnostic imaging suggest that previously undetected CCA may be found in patients undergoing liver transplant (LT) for other indications.

Early experience with LT for unselected CCAs was disappointing with 5-year survival rates ranging from 18% to 25% (9). Recent data suggest that carefully selected patients may do well after transplantation (10). Using the Mayo protocol of aggressive neoadjuvant chemotherapy for small (<3 cm), non-metastatic pCCA, Heimbach et al. found a 5-year survival of 69% in highly selected patients with pCCA, on a background of PSC (11). A subsequent multicentre study involving 12 US LT centres demonstrated a recurrence-free survival of 78%, 65% and 59% at 2-, 5- and 10-years, respectively, using the Mayo protocol (12). A recent Dutch retrospective study looking at 732 consecutive patients with pCCAs (13) identified that only 5% of them were potentially eligible for LT using the same protocol. Recent evidence for iCCA suggests that LT might be beneficial for tumours smaller than 2 cm, when compared to surgical resection. An international multicentre study demonstrated that very early (<2 cm) iCCAs had a 5-year survival of 65% following LT (10).

The purpose of this multicentre study was to retrospectively describe the outcomes after LT in recipients that were diagnosed with CCA incidentally on explant.

## Methods and Materials

### Study Design

LT recipients with previously undetected CCA, occasionally on a background of hepatocellular carcinoma (HCC), subsequently found on explant were identified retrospectively from pathology databases across six LT centres in the United Kingdom. A standardised data proforma was completed at each centre including patient age, gender, pre-LT factors (aetiology for liver transplant, pre-transplant imaging, intervention details, biochemical data), LT factors (date of transplant, biochemical markers, explant histopathology data) and post-LT factors (adjuvant chemotherapy data and survival outcomes). Date of last follow-up or death was used to calculate survival times, including those with short follow-up or death during inpatient stay for LT. There was an absence of a pre-determined minimum follow-up period and therefore we have reported estimated survival rates. CCA was classified by tumour-node-metastasis (pTNM) stage using the American Joint Committee on Cancer and Union for International Cancer Control 8th edition (14) and by three anatomical locations iCCA, pCCA and dCCA. CCAs identified near or within the gallbladder were included in the dCCA group. For iCCA, a further sub-classification of T1a and T1b existed which focused on tumour size. We classed these tumours as T1 since pCCA classification did not include tumour size. All patients had undergone pre-operative cross-sectional imaging with at least one modality: Magnetic resonance imaging (MRI) and/or Computed tomography (CT). No ethical approval was sought as only anonymised, routinely collected clinical data was used and no additional procedures were performed.

### Statistical Analysis

Parametric data were described using mean and range, and non-parametric data were described using median and interquartile range (IQR). *p*-values below 0.05 were considered statistically significant. Survival was analysed with Kaplan-Meier analysis using the Log-rank test to compare groups. Fisher’s exact test was used to compare association between categorical variables. STATA 16 MP (StataCorp. 2019. Stata Statistical Software: Release 16. College Station, TX: StataCorp LLC.) was used for statistical analyses, and Origin Pro 2020 (Origin (Pro), Version 2020. Origin Lab Corporation, Northampton, MA, United States) was used as graphing software.

## Results

### Overall Cohort

97 patients with incidental CCA on explant were identified from six UK LT centres between January 1988 and August 2020. Two patients were excluded due to lack of survival data with the remaining 95 patients included in the final analysis: Birmingham (*n* = 30), Cambridge (*n* = 10), Edinburgh (*n* = 22), Leeds (*n* = 9), Newcastle (*n* = 6), and Royal Free (*n* = 18). LT were performed between January 1988 and August 2020. Median follow-up after LT was 2.1 years (range 14 days–18.6 years). Most patients were male (68.4%), median age was 53 (IQR 46–62), and PSC (61%) was the most common underlying liver disease (Table 1). Few patients had findings on pre-operative imaging that indicated duct dilatation (45.0%) and duct thickening (19.4%). Tumour characteristics including site and stage are summarised in Table 2. Data on adjuvant chemotherapy was only available for 19 patients, summarised in Table 3.

**TABLE 1:** Summary of patient demographics and aetiology of disease (N = 95).

Gender	
Male	65 (68.4%)
Female	30 (31.6%)
Median age (IQR) years	53 (46–62)
Aetiology of liver disease (*n* = 95; %)	
Primary sclerosing cholangitis (PSC)	58 (61%)
Hepatitis C (HCV)	13 (13.7%)
Alcoholic liver disease (ALD)	9 (9.5%)
Cryptogenic liver disease	5 (5.3%)
Non-alcoholic fatty liver disease (NAFLD)	3 (3.2%)
Haemochromatosis	3 (3.2%)
Primary Biliary Cholangitis (PBC)	1 (1.1%)
Secondary Biliary Cirrhosis (SBC)	1 (1.1%)
Auto-immune Hepatitis (AIH)	1 (1.1%)
Recurrent cholangitis	1 (1.1%)
Presence of hepatocellular carcinoma	
Yes	28/95
No	35/95
Unknown	32/95

**TABLE 2:** Tumour characteristics including site and staging (N = 95).

Site	
iCCA	40 (42.1%)
pCCA	42 (44.2%)
dCCA	8 (8.4%)
Unknown	5 (5.3%)
**Stage**	
pT1	24 (25.3%)
pT2	41 (43.1%)
pT3	11 (11.6%)
pT4	9 (9.5%)
Unknown	10 (1.1%)
**Size**	
>2 cm	10/95
<2 cm	9/95
Unknown	76/95
**Lymphatic invasion**	
Yes	9/95
No	16/95
Unknown	70/95
**Vascular invasion**	
Yes	7/95
No	19/95
Unknown	69/95

**TABLE 3:** A summary of chemotherapy for patients where data was available.

Regimen	Cycles	Stage	Site	Dead/Alive	Survival (days)
5-FU	1	—	pCCA	Dead	328
Gemcitabine/Oxaliplatin	6	pT3	pCCA	Dead	40
Gemcitabine	—	pT2	pCCA	Dead	785
Capecitabine	8	pT2	pCCA	Dead	987
Gemcitabine/Oxaliplatin	5	—	—	—	—
Capecitabine	6	pT3	iCCA	Alive	—
Gemcitabine/Cisplatin	5	pT2	pCCA	Dead	356
Doxorubicin (chemoembolization)	—	pT1	iCCA	Dead	2084
Doxorubicin	—	pT1	iCCA	Alive	—
Doxorubicin	—	pT1	iCCA	Alive	—
Doxorubicin	—	pT1	iCCA	Alive	—
Doxorubicin	—	pT1	iCCA	Alive	—
Doxorubicin	—	pT2	iCCA	Dead	328
Doxorubicin	—	pT2	iCCA	Alive	—
Doxorubicin	—	pT2	iCCA	Alive	—
Doxorubicin	—	pT2	iCCA	Alive	—
Doxorubicin	—	pT2	iCCA	Alive	—
Capecitabine	4	pT3	pCCA	Alive	—
Capecitabine	—	pT3	pCCA	Alive	—

### Overall Survival

**FIGURE 1:**
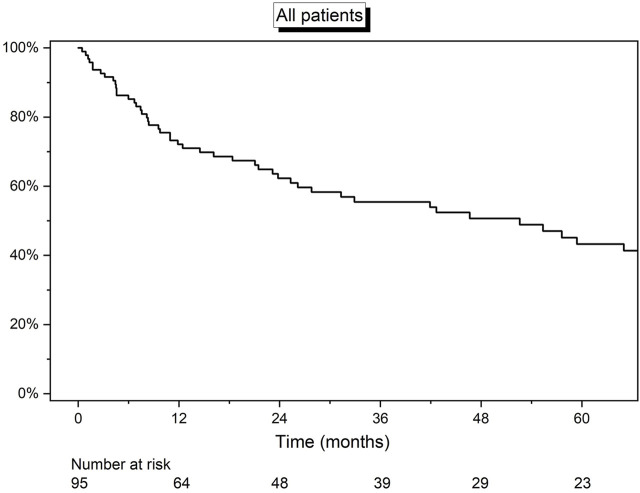
Kaplan-Meier curve detailing the overall survival of all 95 patients.

Overall median survival was 4.4 years (IQR: 0.9–8.4) (Figure 1). At the date of last follow-up (1 August 21), 36 (38%) patients were still alive. The 1-, 3-, and 5-year estimated survival rates were 71.9% (95% CI: 61.5%–79.9%), 55.5% (95% CI: 44.5%–65.4%), and 43.6% (95% CI: 32.0%–54.6%), respectively.

### Survival by Site

**FIGURE 2:**
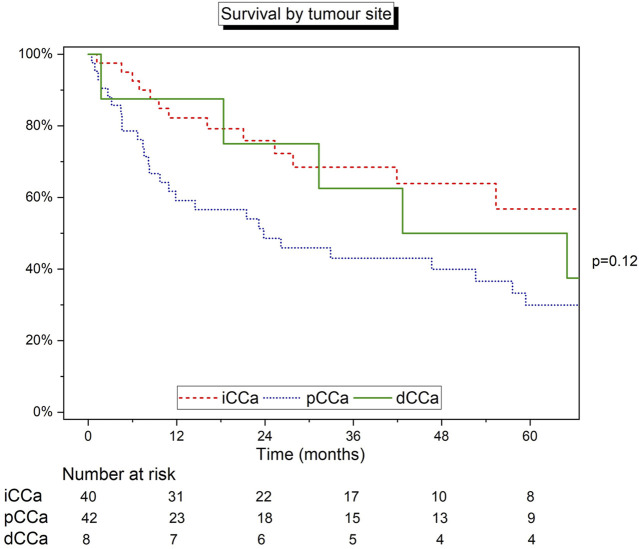
Kaplan-Meier curve detailing the survival of patients grouped by site of tumour. Log‐rank test.

Survival was further analysed by site of tumour, including 90 patients with relevant data available. The median survival was 42.7 months (IQR 18.4–122.6), 68.5 months (25.3–109.7), and 23.8 months (7.4–75.2) for dCCA, iCCA and pCCA, respectively (Figure 2). Survival was lowest in patients with pCCA. Both iCCA (82.1% and 68.7%) and dCCA (87.5% and 62.5%) had similar estimated survival at 1- and 3-years, however, overall 5-year estimated survival was highest in the iCCA cohort (57.1%). There was no statistical difference in survival between the 3 groups (log-rank test *p* = 0.12). After excluding the small group of patients with dCCA, a second log-rank test between iCCA and pCCA was also statistically non-significant (*p* = 0.06).

### Survival by Staging

**FIGURE 3:**
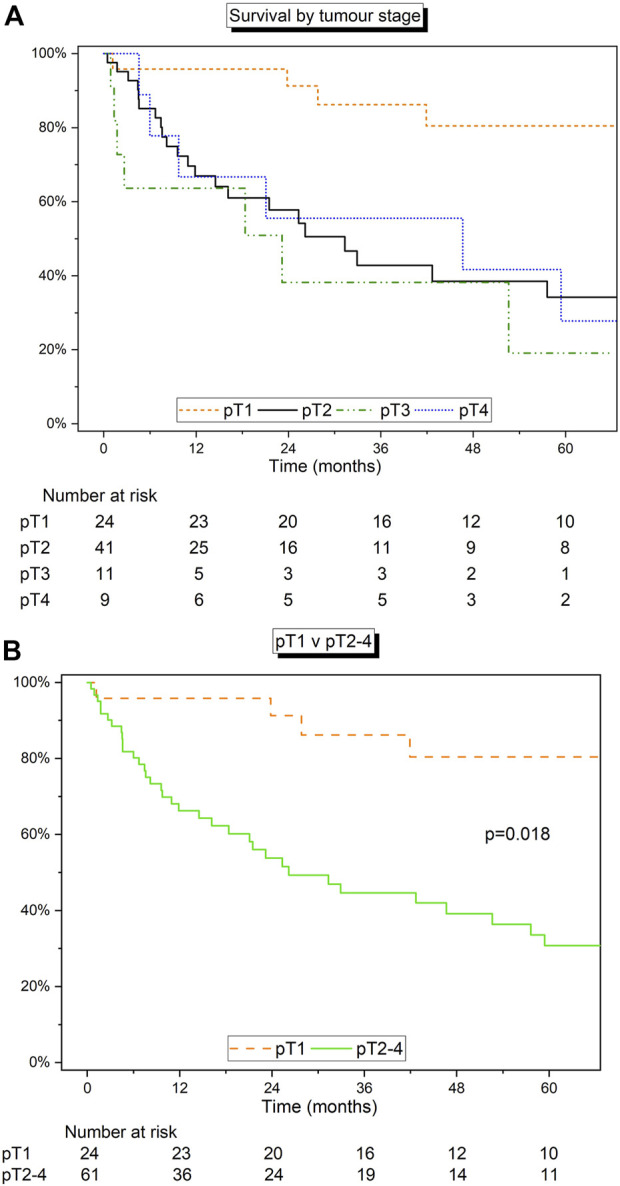
**(A)** Kaplan-Meier curve detailing the survival of patients grouped by size of tumour using pTNM staging. **(B)** Kaplan-Meier curve detailing the survival of pT1 vs. pT2-4. Log‐rank test.

Survival analysis was carried out on patients stratified by pTNM staging. This cohort included 85 patients with pTNM staging information. For pT1 (*n* = 24), pT2 (*n* = 41), pT3 (*n* = 11), and pT4 (*n* = 9), median survival rates were 99.2 months (IQR 69.5–111.8), 31.3 months (8.2–149.5), 23.2 months (1.7–52.6), and 46.6 months (9.7–75.2), respectively (Figure 3A). The 1-, 3-, and 5-year estimated survival rates were highest in the pT1 group. Based on differing survival, pT1 demonstrated relatively superior survival compared to pT2-4 (“other”) staged disease (*p* = 0.018; Figure 3B).

### Survival by Aetiology

**FIGURE 4:**
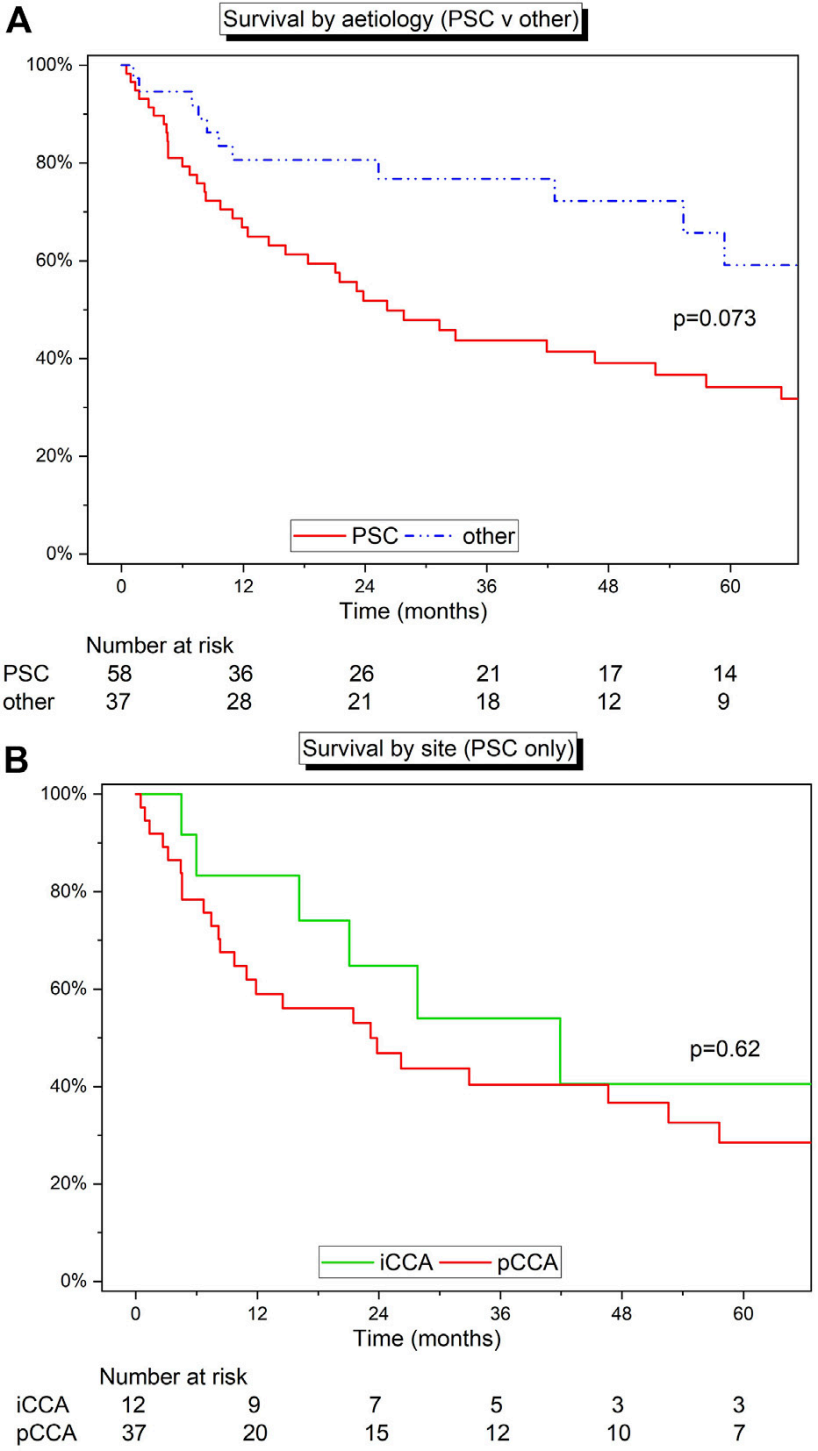
**(A)** Kaplan-Meier curve detailing the survival of patients with PSC *versus* all the other indications for LT. **(B)** Kaplan-Meier curve detailing the survival of patients with PSC grouped by site (iCCA v pCCA). Log‐rank test.

Survival analysis was completed by stratifying by aetiology of liver disease. All 95 patients were considered for this section of the analysis. The cohort was split into two groups: 1) Patients with a diagnosis of PSC (*n* = 58), and 2) patients with any other diagnosis (*n* = 37). Median survival was 26.2 months (IQR 8.2–99.2) for patients with PSC and 69.5 months (42.7–109.7) months for alternate aetiologies (*p* = 0.073) (Figure 4A). Patients with PSC had 1-, 3-, and 5-year estimated survival rates of 66.4% (95% CI: 52.5%–77.1%), 43.5% (95% CI: 30.1%–56.2%), and 34.4% (95% CI: 21.7%–47.5%), respectively, and the non-PSC group had 1-, 3-, and 5-year estimated survival rates of 80.6% (95% CI: 63.5%–90.2%), 76.5% (95% CI: 58.1%–87.7%), and 59.1% (95% CI: 35.9%–76.4), respectively. The stage and site of disease by aetiology can be found in Table 4.

**TABLE 4:** Distribution of patients across the sites and stages stratified by aetiology.

	PSC (*n* = 58)	Non-PSC (*n* = 37)
**Site**		
iCCA (*n* = 40)	12	28
pCCA (*n* = 42)	37	5
dCCA (*n* = 8)	6	2
Unknown (*n* = 5)	3	2
**Stage**		
pT1 (*n* = 24)	13	11
pT2 (*n* = 41)	24	21
pT3 (*n* = 11)	9	2
pT4 (*n* = 9)	7	2
Unknown (10)	5	5

The cohort was further stratified by tumour site within the PSC cohort (iCCA or pCCA) (Figure 4B). Patients with pCCA and PSC (*n* = 37) had 1-, 3-, and 5-year estimated survival rates of 58.3% (95% CI: 40.7%–72.4%), 40.0% (95% CI: 23.8%–55.7%), and 28.8% (95% CI: 14.3%–45.1%), respectively, and patients with iCCA and PSC (*n* = 12) had 1-, 3-, and 5-year estimated survival rates of 82.6% (95% CI: 46.5%–95.3%), 54.4% (95% CI: 22.4%–78.0%), and 42.3% (95% CI: 13.2%–69.4%), respectively (*p* = 0.62).

Finally, patients with PSC and early pCCA (pT1-2; *n* = 22) had 1-, 3-, and 5-year estimated survival rates of 63.6% (95% CI: 40.3%–79.9%), 39.1% (95% CI: 18.9%–58.8%), and 33.1% (95% CI: 14.2%–53.4%), respectively.

## Discussion

In this study of patients undergoing liver transplant who were found to have a previously undetected CCA, we found that small tumours with no vascular or lymphatic invasion (pT1) had much better 5-year survival than larger tumours (pT2, pT3, pT4). pCCAs also had poorer 5-year survival when compared to iCCA, however this was not statistically significant. Within the limits of the size of the cohort, patients with CCA on a background of PSC, tended to have worse medium-term survival when compared to other indications, although this group had more advanced disease.

Cholestatic liver disease is a common indication for liver transplantation with patient and graft survival comparable to other indications. Previously, 5-year survival of patients with PSC undergoing LT has been shown to be up to 83% (15). This contrasts with our cohort with cancer on explant, which demonstrated a much lower median survival of 34.4%. This disparity may exist due to the severity of disease observed in the PSC cohort relative to the non-PSC aetiologies: 30.2% advanced disease (pT3/4) with PSC compared to 12.5% with non-PSC (Fisher’s exact test: *p* = 0.071) and highlights the challenge of detection of even advanced CCA in patients with PSC at the time of LT.

Results from our study indicate that early “low risk” stages of CCA have favourable medium-term i.e., 5-year survival. A Scandinavian study has previously shown that CCAs with a pT2 stage or below had a 5-year survival of 48% (16). We narrowed this group further to include pT1 only and demonstrated better survival at 5-years, which is similar to survival rates of people receiving transplants for HCCs. This finding confirms the need for improved protocols for earlier detection of CCA before LT, particularly in PSC patients, and a UK service evaluation offering LT as definitive treatment for a select group of patients diagnosed with early stages of iCCA and pCCA. It also offers multidisciplinary teams additional information with which to counsel patients currently receiving treatment for hepatobiliary disease.

Accurate diagnosis of CCA type is important since it can dictate whether a patient is selected for surgical (resection or transplant) or conservative (chem (radio)otherapy) management. For example, inclusion into the Mayo protocol for LT in pCCA requires the diagnosis of CCA. However, diagnosing these tumours, both in PSC and non-PSC patients, is often challenging. Pathological confirmation of CCA before therapy was obtained in only 52% (45 out of 87) of PSC patients in the Mayo cohort (11). Pre-treatment pathological confirmation was associated with significantly inferior 5-year survival after start of therapy (50% vs 80%; *p* = 0.001) and after transplantation (66% vs. 92%; *p* = 0.01) in the PSC cohort, when compared with no pathological confirmation (17). From these findings, we could imply that pathological confirmation is more likely in larger, more advanced tumours and that half of the PSC patients from the Mayo cohort may have had low-risk tumours and hence better long-term survival, which is also observed in our cohort with pT1 tumours. A subgroup analysis of early stage (pT1-2) pCCA patients with PSC (*n* = 22) showed 1-, 3-, and 5- year estimated survivals of 63.6%, 39.1%, and 33.1%, respectively, in our cohort. Interestingly, in a retrospective review (18) of European Liver Transplant Registry (ELTR) data from 21 centres between 1990–2010, only 28 (19%) patients out of 249 met the strict selection criteria of the Mayo clinic, and only 5% in a Dutch study of 732 pCCA patients from two centres (13).

Our cohort with pCCAs demonstrated a 5-year overall survival of 30.2%. This was in-line with a recent meta-analysis by Cambridge et al. (19) which reported a 5-year survival of 31.6% in patients not receiving neoadjuvant chemoradio-therapy (NACRT). In this series, 5-year survival increased to 65.1% in patients receiving NACRT. In contrast, in the ELTR series, the 5-year survival without NACRT was 58%, which is comparable to the group that received NACRT. These seemingly conflicting data highlight the need for a multi-centre study to definitively address outcomes for highly selected cases of unresectable pCCA.

When comparing by site of disease, we found a significant difference in 5-year survival between the three sites (iCCA, pCCA and dCCA). After excluding the small dCCA cohort, the difference was not significant, but this might be explained by the underpowered nature of our study. iCCAs trended towards a better survival than pCCAs and due to the limited nature of our data, no causal conclusions can be drawn.

Various chemotherapy regimens were utilised in our cohort, however, the decision to provide adjuvant therapy was a difficult one due to the incidental nature of these tumours on explant. Nonetheless, Gemcitabine with or without platinum based alkylating agents (Cis- or Oxi-platin), 5-fluorouracil (5-FU) and Capecitabine were used in a handful of patients. Due to our small numbers and heterogeneity of the data, we were unable to compare survival across the groups. A recent review of adjuvant therapy by Nara et al. found that Capecitabine (BILCAP trial) had a significant difference in survival compared to controls (20) and has since been adopted in various international treatment protocols. A limitation of the BILCAP trial was the failure to find a difference in survival during intention-to-treat analysis. Furthermore, in a separate group of patients with iCCA, chemoembolization using doxorubicin was performed. Previous studies have commented on the acceptable disease control afforded by this therapy and its efficacy as palliative, rather than curative, therapy (21–23).

This study has limitations. It is a retrospective study and due to the long follow-up period, there existed some variation in the data that was collected. These factors limited the scope of detailed analysis involving the entire cohort. There was a lack of central review of pre-transplant imaging and explant histology, and therefore it was difficult to comment on the presence of concomitant HCC alongside cholangiocarcinoma- survival analysis stratified by HCC was excluded as a result. Furthermore, our data is unable to comment on the rate of misdiagnosed HCC on pre-transplant evaluation that was later diagnosed as cholangiocarcinoma on explant. Due to the evolution of staging criteria, patients with earlier transplants had missing staging characteristics and therefore were unable to be included in stratified analysis. Criteria for staging also differed across different sites of CCA- iCCA classification includes measurements of tumour size whereas pCCA classification is based on spread past the bile duct. Additionally, our study was unable to comment on adjuvant chemotherapy or pre-operative ablation performed on patients, and data regarding imaging and imaging characteristics was incomplete due to inconsistent records across hospital systems. Specific parameters like size of tumour, which have previously been used as grouping criteria in the literature, were missing from some cases (10). As a result of non-standardised follow-up protocol across centres, we were unable to comment on the recurrence and recurrence-free survival of CCA. Nevertheless, our cohort represents the largest group of patients with incidental CCA at explant reported in the literature.

In conclusion, our UK multicentre series of patients undergoing liver transplantation, who were found to have CCA on explant, showed improved survival in earlier stage disease (pT1) and in those with iCCA. The late stage of detection and adverse outcomes in the pCCA patients, particularly those with PSC, highlights the need for improved methods of detecting CCA at the time of transplant assessment and monitoring on the waiting list to avoid undertaking transplants with an anticipated poor outcome. However, these data encouragingly support a planned UK prospective service evaluation of liver transplantation in selected cases of early stage CCA.

## Data Availability

The raw data supporting the conclusion of this article will be made available by the authors, without undue reservation.

## Abbreviations

CCA, cholangiocarcinoma; CT, computed tomography; dCCA, distal cholangiocarcinoma; ELTR, European Liver Transplant Registry; HCC, hepatocellular carcinoma; iCCA, intrahepatic cholangiocarcinoma; LT, liver transplant; MRI, magnetic resonance imaging; NACRT, neoadjuvant chemoradiation therapy; pCCA, perihilar cholangiocarcinoma; PSC, primary sclerosing cholangitis; pTNM, tumour-node-metastasis.
